# Social associations and habitat selection delineate two subpopulations of west coast transient killer whales (*Orcinus orca rectipinnus*) in the California Current System

**DOI:** 10.1371/journal.pone.0325156

**Published:** 2025-11-06

**Authors:** Josh D. McInnes, Andrew W. Trites, Kevin M. Lester, Chelsea R. Mathieson, Lawrence M. Dill, Jeffrey E. Moore, Marilyn E. Dahlheim, Jonathan J. Scordino, K. S. Jasper Kanes, Paula A. Olson

**Affiliations:** 1 Marine Mammal Research Unit, Institute for the Oceans and Fisheries, University of British Columbia, Vancouver, Canada; 2 Pacific WildLife Foundation, Port Moody, British Columbia, Canada; 3 Oceanic Research Alliance, Monterey Bay, California, United States of America; 4 School of Resource and Environmental Management, Simon Fraser University, Burnaby, Canada; 5 Evolutionary and Behavioural Ecology Research Group, Department of Biological Sciences, Simon Fraser University, Burnaby, Canada; 6 Southwest Fisheries Science Center, National Oceanic and Atmospheric Administration, La Jolla, California, United States of America; 7 National Marine Mammal Laboratory, National Oceanic and Atmospheric Administration, Seattle, Washington, United States of America; 8 Marine Mammal Program, Makah Fisheries Management, Makah Tribe, Neah Bay, Washington, United States of America; 9 Ocean Networks Canada, University of Victoria, Victoria, Canada; MARE – Marine and Environmental Sciences Centre, PORTUGAL

## Abstract

West coast transient (mammal-eating) killer whales (*Orcinus orca rectipinnus*) inhabit the California Current off the west coast of North America from southern British Columbia, Canada to southern California, United States. Although genetically distinct from other killer whale populations, observed differences in social associations and habitat use of some individuals suggest that west coast transient killer whales may not constitute a single, socially and spatially discrete population. We analyzed 2,232 georeferenced encounters of photographed transient killer whales collected between 2005 and 2021 from dedicated research ship surveys, small vessel surveys, and opportunistic sightings, to assess their social and population structure in relation to habitat characteristics. Using social network and geospatial analysis software, we identified two socially and geographically independent subpopulations with cohesive social structures—one that frequents shallow nearshore coastal areas (inner coast subpopulation) and a second that primarily inhabits deeper waters along the continental shelf-break and slope (outer coast subpopulation). The inner coast subpopulation (n = 345 photo-identified whales) most commonly occurred in intracoastal waterways and along the shallow coastal margins of the continental shelf where they fed primarily on pinnipeds and small cetaceans within 5 km of shore. In contrast, the outer coast subpopulation (n = 211 photo-identified whales) occurred within 6.1 km of the continental shelf-break and in far offshore waters (e.g., 120 km offshore) near deep submarine canyons and subsurface sea mounts—where they fed primarily on pelagic pinnipeds, oceanic delphinids, and large cetaceans. Our findings demonstrate that the west coast transient killer whale population, though genetically distinct, is structured into two socially and ecologically distinct subpopulations along the west coast of North America. This division underscores the need for conservation efforts to be tailored to their unique ecological and social characteristics.

## Introduction

Transient killer whales (*Orcinus orca rectipinnus*) are distributed throughout coastal and offshore waters of the North Pacific Ocean [1–6]. In the North Pacific, these highly mobile predators occur from southern California to the Bering and Chukchi Sea—west along the entire Aleutian Islands, and into the subpolar waters off Russia, the Sea of Okhotsk, and the coast of Japan [1,2,4–11]. In each of these regions, the ecology and behavior of transient killer whales appears to be adapted to hunting and exploiting a variety of marine mammal species in habitats ranging from shallow rocky and sandy coastal areas to the continental shelf-break and offshore submarine canyon systems [7,12–15].

Throughout this large geographical range, transient killer whales appear to consist of at least six populations based on differences in regional acoustic dialects, genetics, and habitat use. These six populations include: 1) a western population encompassing the Sea of Okhotsk, the Russian Kamchatka Peninsula, and the western Aleutian Islands, 2) the eastern and central Aleutian Islands population, 3) the Bering Sea (Pribilof Islands) population, 4) the Gulf of Alaska population, 5) the AT1 or Chugach population isolated to Prince William Sound and the Kenai Fjords, Alaska, and 6) the west coast transient (WCT) population that ranges from Southeast Alaska to southern California [2,3,5,16–18]. In addition, there is a small sympatric group of transients known to hunt near Unimak Island, Alaska in the spring as gray whales (*Eschrichtius robustus*) cross into the Bering Sea from the Pacific — that do not appear to be genetically related to any other known populations [3,19,20]. Additional populations of transient killer whales may yet be identified, while some of the presumed populations may be further divided into subpopulations and management stocks as more data are gathered about their movements, social structure, genetics, acoustic dialects, and dietary specializations [21].

Of all six transient populations, the WCT population has been the most extensively studied, with early research focused on photo-identifying individuals and documenting their association patterns in the coastal waters of Southeast Alaska, British Columbia, and Washington State [1,22]. Later studies determined that transients from these regions share a distinct mitochondrial DNA haplotype and have an acoustic dialect not found in other transient populations in the North Pacific [23–28]. Other studies reported individuals commonly seen along the open coasts of Oregon and California to occasionally associate with transients further north [1,5,29,30]. As such, the WCT population has been considered to include all individuals occurring between Glacier Bay, Alaska (58.5º N) and San Diego, California (32.7º N). However, the tendency has been to partition and geographically catalog west coast transients according to where distinct social groups are most commonly seen (e.g., Southeast Alaska, British Columbia/Washington, or California) [7,22,31,32].

An assessment of the population status of WCT killer whales by the Canadian government in 2007 concluded there was insufficient information to consider individuals primarily seen south of Washington State in California waters as being members of the population that occurs further north in Canada [33]—despite occasional interactions of individual whales and acoustic similarities. However, a follow up study of transients photo-identified in British Columbia waters described the presence of two putative subpopulations [2]—an inner coast subpopulation of well-known individuals that frequented coastal waters, and an outer coast subpopulation of poorly known individuals that occurred further from shore and comprised a mixture of whales, including some from California. Acoustic analyses of transient killer whales along the outer coast of Washington further identified regional differences in their repertoires, and noted differences in the acoustic presence of distinct transient groups based on different vocal dialects along the continental shelf-break off Cape Elizabeth and at the Quinault Submarine Canyon [27].

In terms of population size, a recent catalogue of WCT killer whales photo-identified in British Columbia coastal waters reported a minimum of 349 whales—most of whom are thought to be from the inner coast subpopulation [31]. Further south, a second catalogue of WCT killer whales reported a minimum count of 155 whales off California and Oregon [5,32]—most of whom were observed in offshore waters and are thought to belong to the outer coast subpopulation. The majority of individuals primarily identified in offshore waters of Oregon and California did not match whales identified in the British Columbia catalogue, but individuals were on occasion documented to associate with one another. Thus, the WCT population appears to total over 500 individuals.

The geographic focus of transient killer whale research along the west coast of North America gives the impression that there is a north/south split or gradient in the distribution of the WCT population, and suggests that some individuals may use waters further from shore based on prey specialization along the continental shelf-break in comparison to individuals that tend to be encountered in coastal waters [2,15]. However, it is unclear whether the population structure of WCT killer whales corresponds to a latitudinal gradient, a longitudinal gradient, or a combination of both. One approach to resolving the population structure of these whales is to use multiple lines of evidence to assess the within-population variability in their social structure, association patterns, distributions, movements, and physiographic or oceanographic differences in their habitat use — as has been done to determine the demographic independence of a number of cetacean populations [21].

The following examines the social structure of the WCT population utilizing the California Current—a transboundary ecosystem extending from southern British Columbia, Canada to southern California, United States. Using detailed photo-identification records and geospatial and social network analysis, we assessed how transient killer whales associate across the coastal and offshore waters between California and British Columbia. Our goal was to determine whether they constitute a single population or distinct subpopulations.

## Materials and methods

### Study area

**Fig 1:**
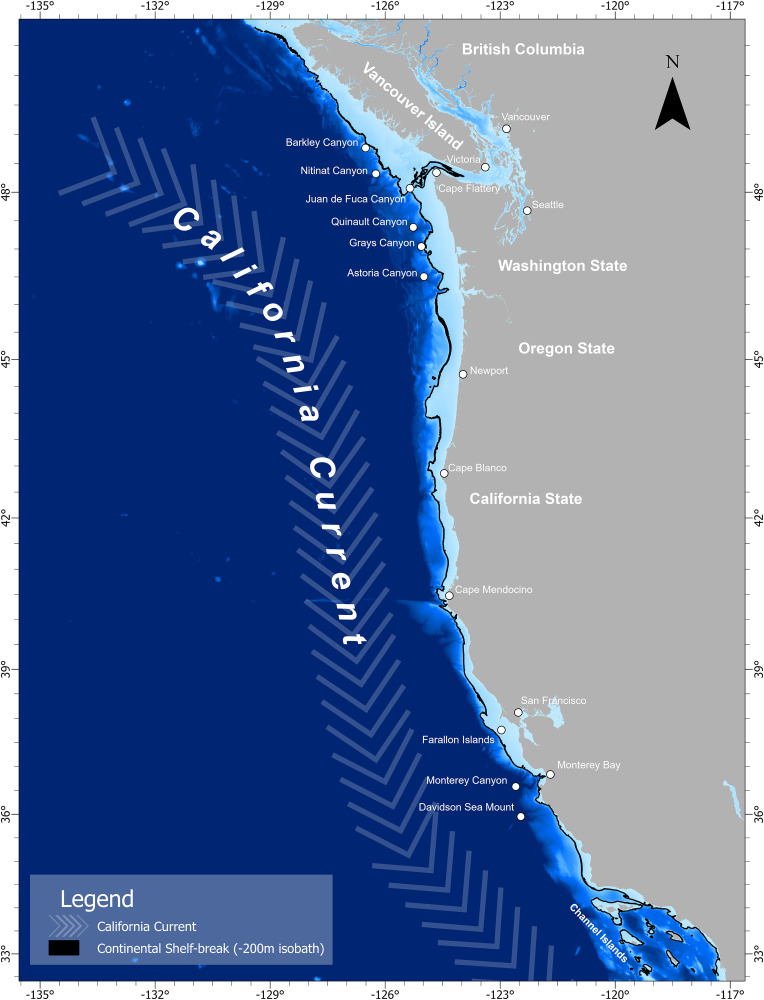
Primary study region encompassing the California Current System. Base map reprinted from NCEI under CC BY license 4.0, with permission from NOAA, original copyright 2022.

Encounters and observations of transient killer whales were recorded from southern British Columbia, Canada (~50.8° N) to southern California, United States (~32.5° N), with survey effort extending seaward up to 556 km offshore (Fig 1). The California Current is a large transboundary current that transports cool nutrient rich water southward along the coast in the upper 500 m of the ocean [34]. In addition, there is also a cold nutrient rich undercurrent that weakly flows north along the continental slope—and an inshore seasonal countercurrent (the Davidson Current) that moves warm temperate waters north during the winter. At the boundary of these currents is a broad oceanic front of oligotrophic waters that extend ~1000 km offshore at the current’s western boundary [34–36]. Along the continental shelf, nutrient rich water is primarily driven by seasonal coastal upwelling brought on by wind circulation patterns that begin in March and continue through August. This complex interplay of water circulation within the California Current Ecosystem creates a dynamic marine environment that supports a wide range of taxonomic species.

The continental shelf, which stretches from southern British Columbia to southern California is a relatively flat plateau that breaks around the 200 m isobath, but narrows latitudinally north to south [37]. The width of the continental shelf ranges from ~50 km off the west coast of Vancouver Island to within 12 km off the central coast of California [37,38]. This variation in continental shelf width, in combination with spatial and temporal variation in oceanographic processes produces significant habitat variation within the California Current [35,39].

To study the habitat-use patterns of WCT killer whales, we divided the study region into four areas: 1) the Salish Sea, 2) the west coast of Vancouver Island, 3) the open Pacific coasts of Washington and Oregon, and 4) the coastal and offshore waters of California (Fig 1).

***Salish Sea***. This inland waterway spans the coastal waters of southern British Columbia, Canada, and U.S Washington State, United States—and includes the Strait of Juan de Fuca, Strait of Georgia, and Greater Puget Sound. The Strait of Juan de Fuca connects the coastal inland waters to the open ocean and is furrowed by the deep Juan de Fuca Canyon that extends from the mouth of the strait into offshore waters. Most of the Salish Sea is dominated by a rocky coast that is glacially incised with shallow inlets, bays, and reefs and is characterized by strong tidal currents.

***West Coast of Vancouver Island***. This area is open to the Pacific Ocean and acts as a transition zone between the rocky coastal Salish Sea and the intermixed rocky and sandy exposed benthic areas along the west coast of Vancouver Island. This area is connected by numerous inlets that extend far inland that provide shelter and habitat for marine mammals and seabirds from the exposed open ocean. It is characterized by a moderately narrow continental shelf off the northwestern end of the island, which gradually widens to approximately 50 km offshore, where it is intersected by a series of submarine canyons (i.e., Barkley, Nitinat) [37,40].

***Washington and Oregon.*** The open Pacific coasts of Washington State and Oregon are dominated by a high-energy system with a relatively uniform continental shelf bathymetry of long stretches of shallow sandy and soft benthic habitats. Peppered through this area are rocky islets that extend just offshore, providing foraging habitat and resting and breeding sites for pinnipeds and seabirds. Along the coast, several human-made breakwater structures built perpendicular to the shoreline protect entrances and maintain navigable channels to coastal rocky intertidal areas, lagoons, estuaries, and river mouths. The continental shelf extends from ~40 km off the Washington coast to ~15 km off Cape Blanco, Oregon (the Cape Blanco upwelling zone) [41]. The continental shelf off Washington is furrowed by the Nitinat, Quinault, Grays, Guide, Willapa, and Astoria canyons, while the Oregon shelf is characterized by a series of shallow banks, including Perpetua, Heceta, and Siltcoos Banks [37,39].

***California.*** The coastal and offshore waters of California act as a major transition zone for many species, with more northern cold temperate species occurring off central and northern California, and warm temperate species frequenting southern California [42,43]. This region is dominated by a narrow continental shelf and a steep continental slope that promotes coastal upwelling and brings deep oceanic waters close to shore [37,44]. The seafloor is dominated by a number of complex geomorphologic features including seamounts (i.e., Davidson, Guide, and Pioneer), submarine canyons (Monterey, Bodega, Pioneer, and Lucia), the offshore Farallon Islands, and the bathymetrically convoluted Southern California Bight and associated Channel Islands [37,38,44,45].

### Data collection

The California Current Killer Whale Study (CCKWS), a sub-study of the Oceanic Research Alliance (ORA), has conducted research surveys and curated data on transient killer whales since its inception in 2005 to promote objective science-based research into the ecology and behavior of killer whales in the North Pacific. This database includes information collected from a variety of sources, including data from dedicated research ship surveys, small vessel surveys, and opportunistic sightings.

**Fig 2:**
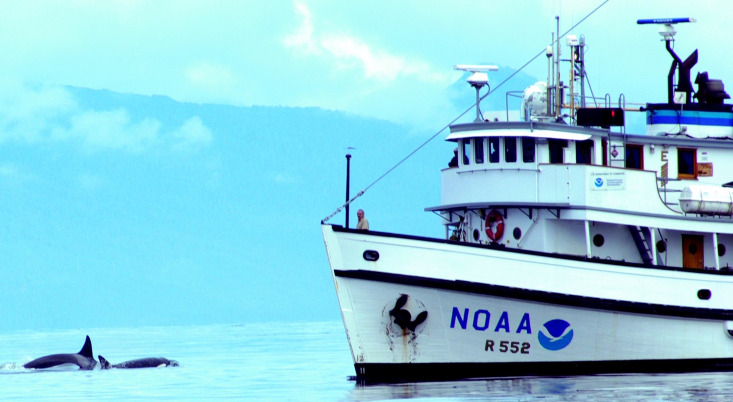
A NOAA research vessel with transient killer whales in protected coastal waters. Photograph by Marilyn Dahlheim, NOAA National Marine Mammal Laboratory.

***Large research ship surveys.*** Large ship surveys were conducted by the Southwest Fisheries Science Center/National Marine Fisheries Service/National Oceanic and Atmospheric Administration (SWFSC/NMFS/NOAA) using the NOAA vessels *McArthur*, *McArthur II*, *David Starr Jordan*, and *Reuben Lasker* (52–64 m) for marine mammal stock-assessments in 2008, 2009, 2014, 2015, and 2018 [17,46–51]. Surveys through 2015 were conducted along a grid of planned transect lines, placed systemically throughout the survey area, which spanned 556 km offshore the US west coast—from southern California to southern British Columbia, Canada [50]. The 2018 survey differed in extent and objectives from previous surveys by using parallel transects (rather than a grid) that were mostly spaced 10–37 km, and were largely limited to continental shelf waters [52]. The primary protocol followed visual line transect methodology, whereby vessels travelled at ~18 km/hr along transects, and observers used binoculars to identify and systematically count animals [46]. Photoidentification and behavioral data collection of transient killer whales were conducted opportunistically from the ship, or from small boats launched from the larger vessel to pursue groups of whales to obtain observations of predation events, identification photographs, and biopsy samples (Fig 2).

***Small vessel surveys.*** Small vessel surveys were primarily focused on collecting information on the behavioral ecology, photoidentification, and fine-scale habitat use patterns of transient killer whales and were conducted regularly by 2–6 observers in 8–12 m vessels from April through November 2005–2021. Small vessel surveys were conducted by the Oceanic Research Alliance off southern Vancouver Island, by Makah Fisheries Management along the outer coast of Washington, by Oregon State University’s Marine Mammal Institute off the Oregon coast, and by Marine Life Studies and the Oceanic Research Alliance off the central coast of California. Surveys off southern Vancouver Island primarily left from Victoria and Pedder Bay, BC and were focused on searching the coastal waters of the Strait of Juan de Fuca. Surveys along the Washington coast primarily covered the western portion of the Strait of Juan de Fuca, along the outer coast, and the offshore waters of Swiftsure Bank—with transients being opportunistically encountered during dedicated studies of gray whales (*Eschrichtius robustus*) and Steller sea lions (*Eumetopias jubatus*) [53–55]. Dedicated killer whale surveys conducted off the central coast of California were operated out of Moss Landing and primarily focused effort in Monterey Bay, but also extended south to Big Sur and north to Santa Cruz [15,32]. Lastly, observations of transient killer whales were also recorded and compiled from experienced naturalists aboard whale ecotour vessels throughout the entire study area.

Transient killer whales were located visually by scanning the water from the vessel or from an elevated point from shore using handheld 7 x 50 binoculars. An encounter was defined as any period of observation that was > 10 minutes in duration, and where each individual whale was photographed, and their behavior was noted. Data recorded during an encounter included the date, location (latitude and longitude), time (24-hour clock), group size, direction of travel, dive durations, estimated distance between individuals, and any active surface behaviors. The locations of whales were recorded using a global positioning system (GPS) to track the movements of whales in relation to different habitat features (i.e., shallow coastal shorelines, pinniped haul outs, deep-sea canyon contours). Predation events were recorded when a group or individual whale was observed actively pursuing or feeding on prey, and typically involved extensive prey handling or visible prey parts in the mouths of whales or at the sea surface (e.g., bits of blubber, muscle tissue, internal organs, blood, oil slick). Some encounters were prematurely terminated due to adverse weather conditions, fuel constraints, approaching darkness, or if the whales were lost visually.

All small vessel surveys and whale watch ecotours in the coastal waters of British Columbia adhered to regional regulations and guidelines for observing killer whales. These guidelines primarily required maintaining a distance of at least 200 meters from the whales, allowing researchers and naturalists to continue to capture clear photographs for individual identification and to observe and classify whale behavior. Observations of transient killer whales during NOAA SWFSC ship surveys were conducted under NOAA NMFS permit #22306. Research surveys conducted by marine mammal scientists with Makah Fisheries Management in Washington State’s coastal and offshore waters were carried out under NOAA NMFS permits #21348, 781–1824, 782–1438, 782–1719, and 14245. Similarly, research off the Oregon coast by Oregon State University’s Marine Mammal Institute was authorized under NOAA NMFS permits #14856 and 21585. In California waters, Marine Life Studies conducted research under NOAA NMFS permits #1094–1836, 15621, and 20519, with certain encounters occurring under Marine Mammal Protection Act permit #782–1510 (Principal Investigator: Marilyn Dahlheim).

***Opportunistic sightings***. Opportunistic “presence only” sightings of transient killer whales were compiled from 2005 to 2021 throughout the California Current System. Data sources included: 1) public sightings collected year-round that were emailed or shared via a number of social media platforms, 2) firsthand and historical reports from experienced researchers, naturalists, and long-term contributors (e.g., Strawberry Isle Marine Research Society, Discovery Whale Watch), and 3) reports compiled from open-access public sighting databases (e.g., INaturalist, Orca Network, Wild Ocean Whale Society). Each sighting used was followed up with an interview, and information was collected on the date, time, location (latitude & longitude), and photographs were collected to confirm group/individual whale identifications.

***Data standardization.*** We consolidated all records from the above sources into single “daily encounters” to determine the presence/absence of each transient group and individual whale for 2005–2021. An “encounter” was defined as any period of time that transient killer whales were documented, and all individuals in a social group could be photo-identified at a particular georeferenced location on a given day. To minimize effort bias, we created a relative effort layer in a geographic information system framework (see GIS Analysis below) and analyzed sightings within each of the four study areas (Salish Sea, west coast of Vancouver Island, Washington/Oregon, California) [56]. Finally, if a particular photo-identified group or individual whale was reported by multiple sources on a given day, only the first initial sighting location was used for that data point. This procedure standardized each data source, regardless of survey methodology, to reflect only parameters that can be used to address association patterns of individual whales and the locations of encounters with these individuals in relation to specific habitats.

### Photoidentification

During an encounter with transient killer whales, dedicated effort was made to photograph all individuals in the group using high quality DSLR cameras with telephoto lenses (100–400 mm). Photographs from experienced naturalists, field researchers and citizen scientists, were only used if the reported group size exactly matched the number of whales photographed during that observation period.

**Fig 3:**
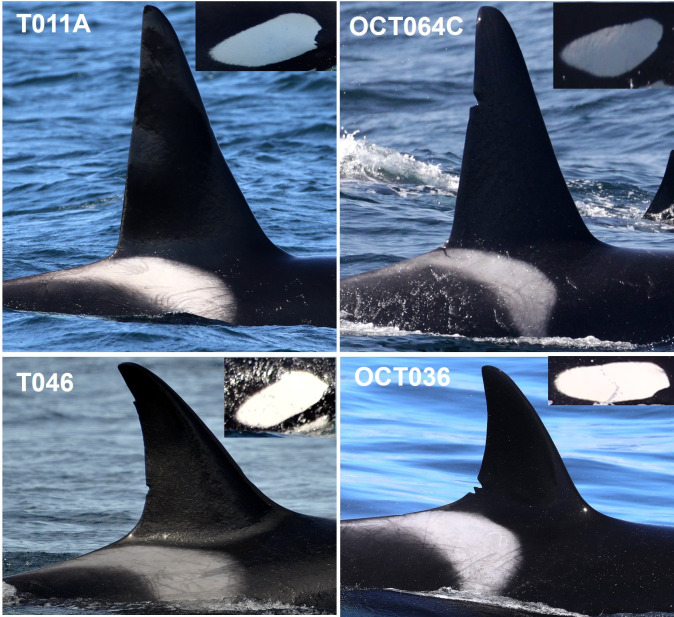
Photoidentification images of west coast transient killer whales collected in nearshore and offshore waters of the California Current. Top photographs are of adult males T011A (inner coast) and OCT064C (outer coast) and the bottom photographs are of adult females T046 (inner coast) and OCT036 (outer coast). Photographs by Josh McInnes UBC MMRU.

Individual whales were identified using at least two unique markings, including notches or nicks on the dorsal fin, scarring on the saddle patch, and the shapes of the dorsal fin, saddle patch and post-ocular patches [8,57] (Fig 3). WCT killer whales encountered in the study area were cataloged and assigned alphanumeric designations (e.g., T011A, OCT090), and confirmation of identification and group membership was made by comparing photographs to published catalogues [5,22,31,32] and an unpublished database of images maintained by the Oceanic Research Alliance (ORA) and the Marine Mammal Research Unit (MMRU), at the University of British Columbia’s (UBC) Institute for the Oceans and Fisheries (IOF).

Photographs were graded for the best left and right sides of the dorsal fin and post-ocular patches for each whale based on focus, angle, light, and distance. Identification photographs were updated annually for as many individuals as possible to track changes in saddle patch markings and physical structure of the dorsal fin. On occasion, individual whales could not be photographically matched, but were observed in association with known WCT killer whales. These encounters with previously undocumented whales primarily occurred during offshore surveys in remote parts of the study region. In this situation, a new alphanumeric photo-identification number was created, and the individual was subsequently catalogued [5].

### Transient killer whale social structure

**Fig 4:**
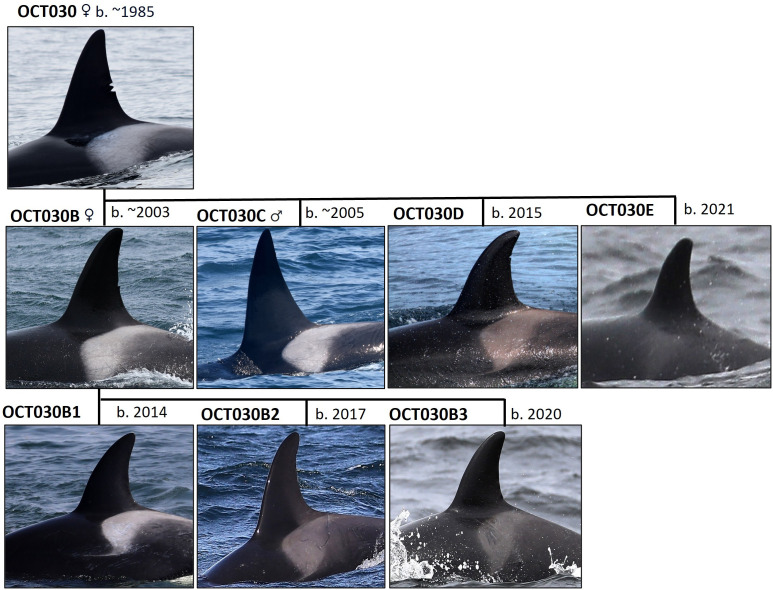
Genealogical schematic of the transient killer whale OCT030 matrilineal group. **Photographs show identities and sex (if known) for each whale as of 2021.** Representation of matrilineal descent is based on studies and methods described in [5], and identification images are by Josh McInnes UBC MMRU.

The social organization of transient killer whales is centered around the strong long-term associations between adult females and their offspring (Fig 4). Population structure has been primarily described as ‘fission-fusion’, which is characterized by loose associations of individuals within the population [13,58,59]. Transient maternal groups typically range in size from 2 to 10 individuals and contain whales of both sexes and all ages—with dispersal occurring for some adult male and most female offspring at sexual maturity [13]. Male dispersal appears to result from the death of their post-reproductive mothers, or when maternal groups produce more than one male offspring, resulting in either male offspring becoming nomadic (roving males) and associating for short periods of time with various other transient groups or individual whales. Female offspring primarily disperse after the birth of their first offspring, and tend to be highly gregarious by associating with other maternal groups for extended periods of time [58].

We defined a maternal group as an adult female and all of her offspring that have not been known to have dispersed. Single adult males were defined as their own group if they were observed independent for >50% of sightings from any particular known matriline. Finally, a social group encounter was described as a period when multiple maternal groups associated or interacted, with formations typically occurring during periods of foraging and socializing.

### Analysis of social structure

Photoidentifications of encountered transient killer whales were used to investigate the broad social structure of the WCT population. Individual whales were categorized as members of the population based on long-term monitoring studies that linked individuals through association patterns [1,22,31,32]. For example, if whale A was observed with whale B, and whale B with whale C, all three whales were considered to be members of the same population through intermediate associations [1]. Because transient killer whales exhibit fluid association patterns, social structure and habitat use was analyzed at the individual level, with whales being considered associated when they were observed in the same group during each encounter (gambit of the group; implies that each individual in a group is associating with every other individual in that group, per [60]). To minimize spurious associations, analysis was conducted only on individuals identified at least three times and where all individuals in the group were photo-identified [61].

Association data among individual transient killer whales was analyzed using SOCPROG 2.9, a software program developed in MATLAB 5.1 (The Mathworks, Inc., Natick, MA) [62]. Because the primary goal of the study was to analyze social structure at the population level, and not between conspecifics in a social group (i.e., dyads), all individual transients were included and the association strength between members was calculated by computing pair-wise association indices (AI), using the simple radio index (SRI) [63]. The SRI gives a weighted network of associations based on how often individuals are seen together versus apart. This particular measure is defined as:


$${\;SRI}_{ij}=\;\frac{X_{ij}}{X_{ij}+Y_{i}+\;Y_{j}\;}$$ (1)

where $X_{ij}$ is the number of observed transient groups in which whale i and whale j were seen together, $y_{i}$ is the number of transient groups in which whale i was observed, but not whale j, and $y_{j}$ is the number of transient groups in which whale j, was sighted but not whale i. The values of the SRI range from 0 (never associated) to 1 (always associated).

### GIS analysis

Geographic information system (GIS) was utilized to quantitively describe the distribution and habitat use patterns of different photo-identified groups or individual whales observed based on common physical data for the entire study area. We used water depth (m), distance to the continental shelf-break (km), and distance to shore (km) to describe differences in large and fine-scale habitat use patterns of individual transient killer whales. Analyses of these physiographic and oceanographic variables enabled further inferences into how particular environmental features may influence the spatial ecology and association patterns of transient killer whales.

***Data and projection.*** The geospatial locations of encounters with transient killer whales were recorded using decimal degree (DD) coordinate value data. This data were pre-processed in Microsoft Excel and cleaned of grammatical errors in preparation for import into the Environmental Systems Research Institute’s (ESRI) ArcGIS Pro (version 3.2.0) software. This flat file spreadsheet data was then converted to a point feature layer and projected using the World Geodetic System into the appropriate UTM Zone (WGS) 1984.

***Water depth calculation.*** Water depth is a highly variable measure that differs substantially between and within areas and is dependent on oscillations in complex tidal cycles. To account for such differences in regional tidal height, the mean high water (MHW) bathymetric datum downloaded from NOAA’s National Centers for Environmental Information (NCEI) bathymetric data viewer in a raster form at a 90-meter resolution was used. For the geomorphologically complex intracoastal waterways of the Salish Sea, a digital elevation model (DEM) at a resolution of 30 meters was used to describe water depth contours of relatively shallow straits, inlets, and bays. Water depth was calculated based on observation point data projected over NOAA DEM raster data and processed using the *extract multi values to points tool* in ESRI ArcGIS Pro. The process yielded a new data field that displayed the average depth of the water column underneath each observation point within 30–90 meters of each point location.

***Definition of the continental shelf.*** For the purposes of our study, the continental shelf extended from the nearest shore to the 200 m isobath line that marks the beginning of the continental shelf break, which extends to the continental slope in the 500 m depth isobath [37,38]. To calculate the location of the continental shelf-break, a 90 m resolution DEM dataset that was processed using the *extract by attributes tool* in ArcGIS to select for water depth values of 200 m was used. Because the width of the continental shelf varies considerably from north to south, the width for the shelf for each observation point was calculated from southern British Columbia to southern California. The NOAA DEM raster data was extracted to form a line indicating the 200 m isobath and another line to indicate the shoreline. The 200 m isobath line feature was processed using the *generate points along the line tool* to create a point feature for every 3 km. This new point layer was then processed using the *near tool* to calculate the distance from each point to the nearest shoreline, the results of which indicated the width of the continental shelf. Continental shelf width information was then transferred to each observation data point using the *spatial join tool* and then averaged by region using the *summarize within tool* for each regional polygon.

***Distance from the continental shelf-break.*** To calculate the navigable water distance from each observation point to the continental shelf-break, the 200 m isobath raster data layer was extracted from the NOAA DEM dataset using the *integer tool*, then converted to a polygon feature layer. The NOAA DEM dataset was processed two more times using the *extract by attributes tool* for values of at least 1 m elevation, indicating areas of land, and values of at most 1 m elevation for areas of water. The new land-indicating raster layer was pre-processed using the *integer tool*, then converted to a polygon layer for later use. We then processed the new water-indicating raster layer using the *distance accumulation tool*, which accounted for barriers by implementing the previously mentioned land-indicating polygon to measure the distance to the continental shelf-break for each 30 m square around land barriers. This was useful for dealing with distance around land barriers in the convoluted Salish Sea. Each 30 x 30 m cell of the new distance accumulation raster was then converted to a point feature using the *raster to point tool*. This yielded an array of point data containing information on the water distance to the continental shelf break, which was then transferred to each observation location via a spatial join.

***Distance from shore calculation***. To calculate each observation’s distance from shore, a shoreline feature layer was derived from NOAA MHW DEM at 1 m elevation using the *extract by attributes tool* in ArcGIS. This process yielded an approximate shoreline from which each observation point was measured using a spatial join, thereby creating a new field that displayed distance from the nearest shore in meters.

***Effort density calculation***. The distribution of effort across the study area was visualized using the *create fishnet tool* to generate a grid of 5 km polygons. Effort data in the form of lines, such as GPS tracks of research vessels, were processed using the *generate points along line tool* to create a new point layer in which a point feature was created every 3 km of search distance. Opportunistic sightings and track line points were then combined using the *append tool*. The number of points contained within each fishnet polygon were then counted using the *summarize within tool,* which generated a matrix of relative effort across the study area.

### Habitat and spatial predictors of killer whale associations

A Multiple Regression Quadratic Assignment Procedure (MRQAP) was used to examine how location and environmental factors may have influenced the frequency with which individual whales interacted or associated with each other. MRQAP was used to evaluate how well the predictor matrices—which included habitat similarity, geographic location (latitude and longitude), and matrilineal membership—accounted for variation in the response matrix (i.e., killer whale association rates). This analysis tested whether associations between whales reflected a north-south distribution of individuals, as well as the extent to which associations between individuals corresponded to habitat differences related to water depths, distance to the shelf break, and distance from shore.

The MRQAP was run using the netlm() function from the *sna* R package [64] with predictor matrices that included latitude and longitude, distance from shore, water depth, and continental shelf similarity—and a response matrix of pairwise association rates. Statistical significance was determined using 1,000 QAP permutations, which accounted for the non-independence of network data due to autocorrelation.

## Results

### Survey effort

**Fig 5:**
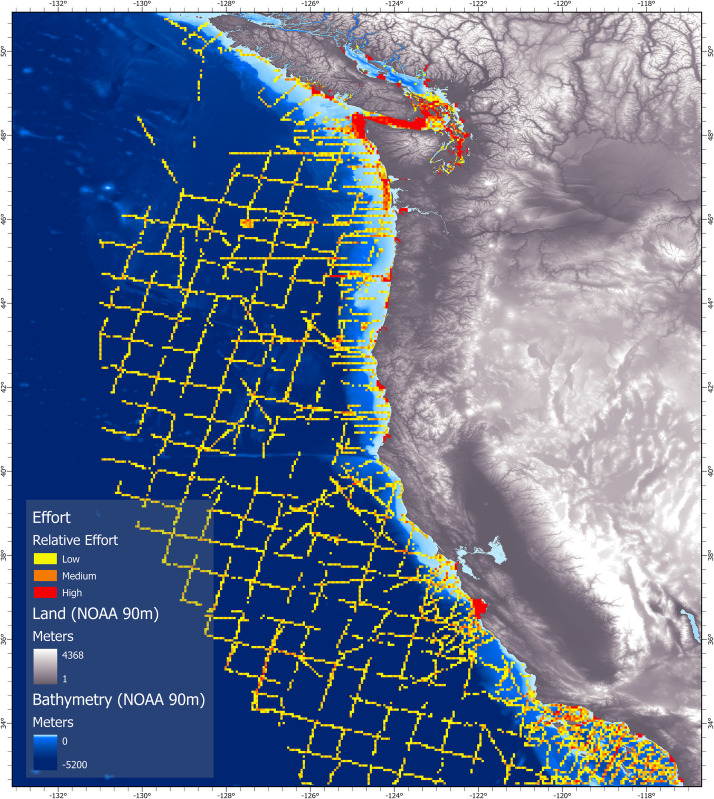
Relative survey effort compiled from NOAA SWFSC ship surveys, small vessel surveys, and opportunistic encounters from 2005-2021. Effort is shown according to a grid of cells (5 x 5 km), with red shading representing an index based on the number of times each cell was surveyed. Base map reprinted from NCEI under CC BY license 4.0, with permission from NOAA, original copyright 2022.

WCT killer whales were encountered 5,456 times from 2005–2021 during research ship surveys, small vessel surveys, and opportunistic sightings in the waters of the California Current. Of these encounters, 2,232 had geospatial locations and photographs to identify all individuals present (Table 1). Transect lines surveyed by NOAA research ships totaled 49,180 km over 335 days of search effort between southern British Columbia and southern California (Fig 5). Killer whales were observed during 43 encounters, of which 12 encounters included individuals that were photo-identified as members of the WCT population (Table 2). These 12 encounters occurred throughout all years surveyed (2008: n = 2, 2009: n = 3, 2014: n = 2, 2015: n = 2, 2018: n = 3). A total of 852 small-vessel surveys covering a distance of 37,424 km had 262 encounters with WCT killer whales (Fig 5). Of these encounters, 148 occurred in the Salish Sea, 9 were along the open coast of Washington and Oregon, and 105 occurred in the coastal and offshore waters of California (Table 1). In contrast to research ship and small vessel surveys, opportunistic sightings covering the entire study area provided the largest source of information on the spatial distribution, movements, habitat use, and association patterns of WCT killer whales. They yielded 1,957 photo-identified encounters of which 78% (n = 1,517) were in the Salish Sea, 4% (n = 80) occurred off the west coast of Vancouver Island, 6% (n = 110) were off the open coast of Washington and Oregon, and 13% (n = 250) were documented off California.

**Table 1:** Encounters with west coast transient killer whales in the California Current System.

Area	Years	Survey type	Inner coast encounters	Outer coast encounters
Salish Sea	2005-2021	Opportunistic sighting	1484	33
	2005-2021	Small-vessel survey	144	4
West Coast Vancouver Island	2015-2021	Opportunistic sighting	79	1
	2015, 2018	Research-ship survey	1	1
Washington and Oregon	2013-2021	Opportunistic sighting	106	4
	2005-2021	Small-vessel survey	9	0
	2008, 2014, 2015, 2018, 2021	Research-ship survey	1	2
California	2006-2018	Small-vessel survey	0	105
	2006-2021	Opportunistic sighting	9	241
	2008, 2009, 2014, 2015, 2018	Research-ship survey	0	8

**Table 2:** Number of west coast transient killer whale encounters during five years of NOAA Southwest Fisheries Science Center (SWFSC) marine mammal stock assessment surveys in the California Current System. Data were collected in Beaufort Sea states of <5.

Year	Source	Research Ship (m)	Survey Name	Region	Effort (km)	Inner Coast encounters	Outer Coast encounters
2008	SWFSC	NOAA *McArthur II* (62 m)	ORCAWALE^1^	CA, OR, WA, BC	11,600	0	2
2009	SWFSC	NOAA *McArthur II* (62 m)	DELPHINUS^2^	CA	3,566	0	3
2014	SWFCS	NOAA *Ocean Starr* (52 m)	CalCurCEAS^3^	CA, OR, WA, BC	9,330	0	2
2015	SWFSC	NOAA *Reuben Lasker* (63 m)	CLAWS^4^	CA, OR, WA, BC	1,278	1	1
2018	SWFSC	NOAA *Reuben Lasker* (63 m)	CCES^5^	CA, OR, WA, BC	9,915	1	2

**Oregon, California and Washington Line Transect and Ecosystem**^**1**^, **Ecosystem Survey of Delphinus Species**^**2**^, **California Current Cetacean & Ecosystem Survey**^**3**^, **Collaborative Large Whale Survey**^**4**^, **California Current Ecosystem Survey**^**5**^

### Photo-identification

Analysis of photographs resulted in 13,152 individual identifications of WCT killer whales from 159,844 photographs taken during encounters. Analyses of the left-and right-sides of the dorsal fin, saddle patch, and post-ocular patches, resulted in a minimum of 556 different individuals, comprising 117 matrilineal groups, 46 nomadic males, and 19 single/or post reproductive females. Of these whales, 63% (n = 351 whales) were photo-identified in the Salish Sea, 15% (n = 86 whales) were identified off the west coast of Vancouver Island, 22% (n = 124 whales) were identified off the open coast of Washington and Oregon, and 42% (n = 234 whales) were identified in coastal and offshore waters of California (Table 3). Photographic re-sightings revealed movements of 167 whales between two areas, 62 whales between three areas, and 3 whales between all four areas (Table 3). The number of sightings per individual whale ranged from 1 to 235 (median ± SE = 8 ± 1.5) with 85% of individuals being re-sighted.

**Table 3:** Numbers of photo-identified west coast transient killer whales seen by region and the number of regions they were seen from 2005-2021. The shaded cells identify the combination of regions where inner and outer coast transients were counted—and show the number of individuals that used just one region or as many as all four regions. Numbers of outer coast transients are shown in italics.

Number of areas used	Region				Population	
	Salish Sea	W Coast Vancouver I.	Washington & Oregon	California	Inner	Outer
1					136	*5*
1 1 1					4	*0*
1 1					12	*5*
1					5	*157*
2					51	*0*
2					4	*0*
2					0	*13*
2					14	*22*
2					2	*1*
2					60	*0*
3					31	*0*
3					1	*1*
3					23	*6*
3					0	*0*
4					2	*1*
Total	317/ *34*	83/ *3*	110/ *14*	45/ *189*	345	*211*

Group size during encounters ranged from 1 to 34 whales, with the most commonly observed group size being 4 (mode = 4, $\bar{x}$ ± SE = 5.73 ± 0.08, n = 2,232 encounters). For the 117 matrilineal groups for which the number of individuals present was available (using long-term observations from this study and published catalogues), maternal group size (≥2 whales) ranged from 2–10 whales, with the most commonly observed being 4 (mode = 4, $\bar{x}$ ± SE = 4.17 ± 0.17, n = 117 matrilineal groups).

### Social network analysis

**Fig 6:**
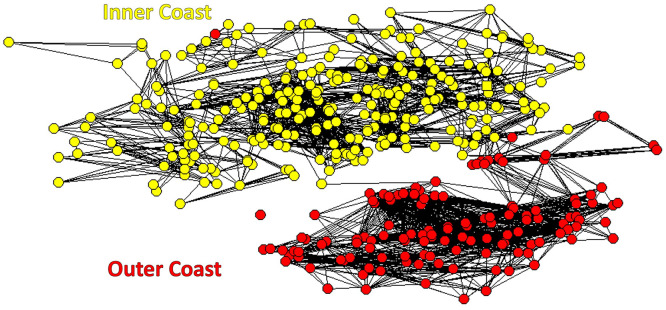
Sociogram showing individual associations between 405 photo-identified west coast transient killer whales encountered >3 times. Direct associations (seen together at least once) are indicated by solid lines, and the length of each line indicates the strength of the association based on the SRI. Inner coast whales (n = 272 individuals) are shown with yellow circles, and outer coast whales (n = 133 individuals) with red circles.

A social network analysis was conducted for 405 whales, excluding 151 individuals that were encountered fewer than three times from a minimum of 556 photo-identified WCT killer whales (Fig 6). The cophenetic correlation coefficient value of the hierarchical cluster analysis was 0.97, which indicates a dichotomy in social structure [60]. This resulted in a maximum modularity index (Qmax = 0.78), which occurred when the photo-identified individuals were clustered by the network analysis into at least two cohesive clusters [65]. One cluster consisted of at least 272 whales that were strongly associated with coastal nearshore environments (i.e., 67% of 11,000 identifications from 1,828 encounters), and a second cluster of at least 133 whales that were photo-identified primarily in offshore waters (i.e., 33% of 2,152 identifications during 404 encounters). These clusters were therefore classified as ‘inner’ and ‘outer’ WCT killer whales consistent with naming proposed by [2].

### Distribution and large-scale habitat use

**Fig 7:**
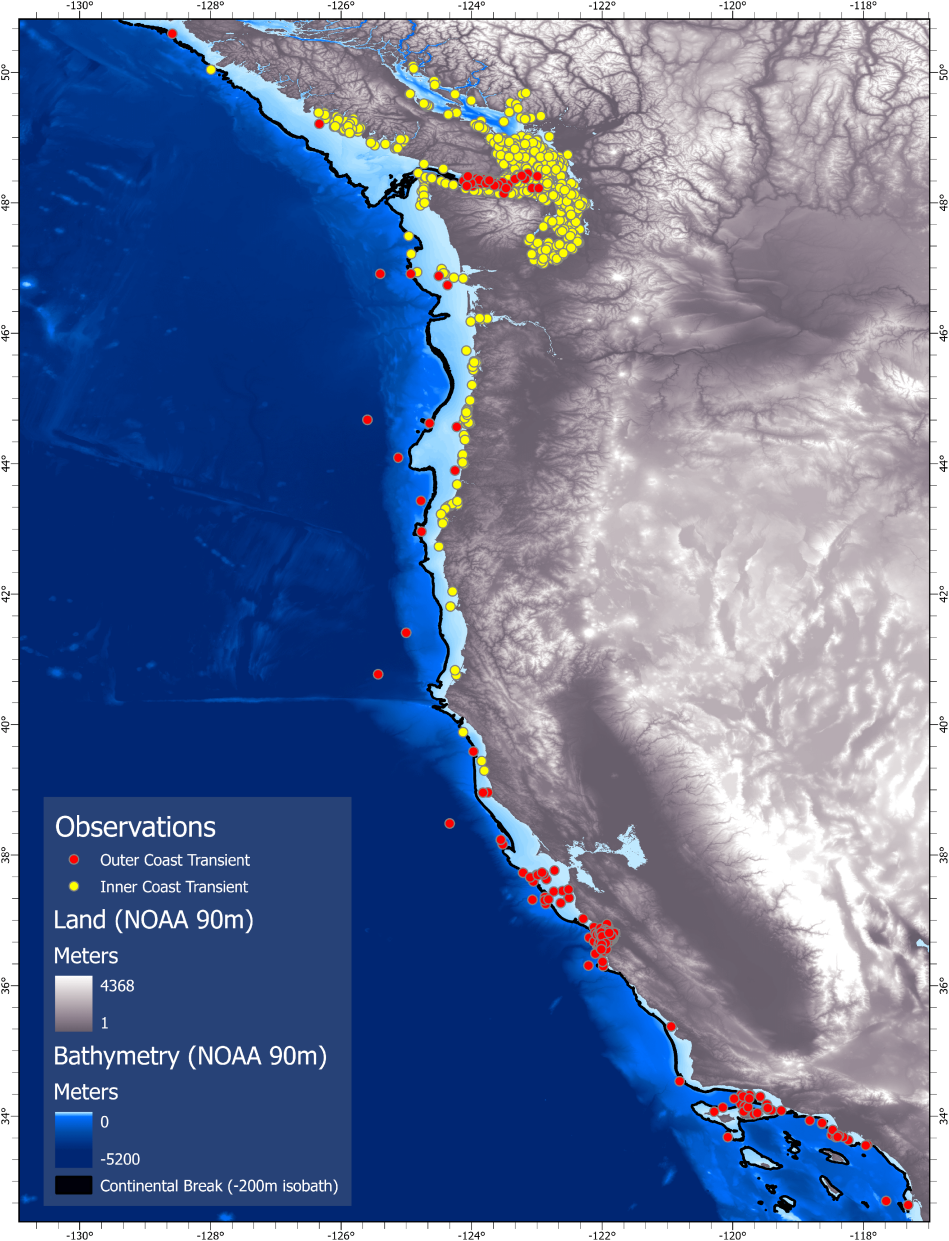
Spatial distribution of encounters with west coast transient killer whales in the California Current System (2005-2021). Encounters are identified by subpopulation (yellow–inner coast, n = 1828 encounters; red–outer coast, n = 404 encounters). Base map reprinted from NCEI under CC BY license 4.0, with permission from NOAA, original copyright 2022.

Within the California Current System, encounters with WCT killer whales occurred from the northwest coast of Vancouver Island (50°02’ N) to as far south as southern California (32°31’ N). Individuals encountered across this broad range were not randomly distributed, but showed two distinct large-scale patterns of habitat use. Most notably, the inner coast transients (n = 345 whales) were primarily encountered in protected intracoastal waterways and along the coastal margins of the continental shelf from the Salish Sea (50°5’ N) to Monterey Bay, California (36°34’ N) — while the outer coast transients (n = 211 whales) were widely distributed in offshore waters, particularly near the continental shelf break and slope from the northwest coast of Vancouver Island (50°35’ N) to as far south as San Diego, California (32°38’ N) (Fig 7).

**Fig 8:**
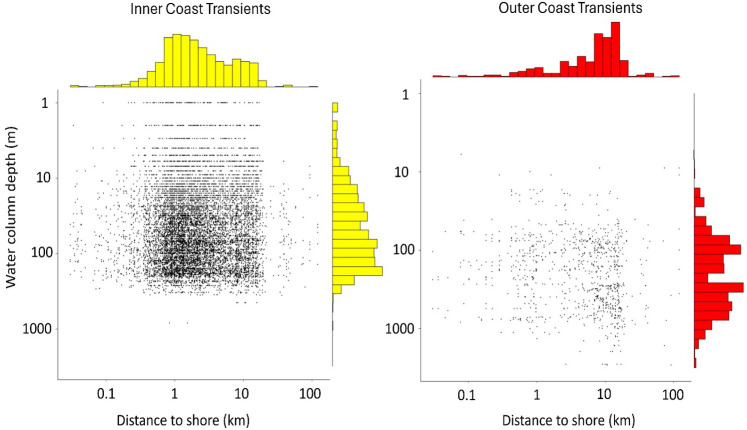
Marginal distributions of the distance to nearest shoreline relative to water column depth for recorded encounters with inner and outer west coast transient killer whales. Data are log transformed (n = 11,000 inner coast, and n = 2,152 outer coast identifications).

**Fig 9:**
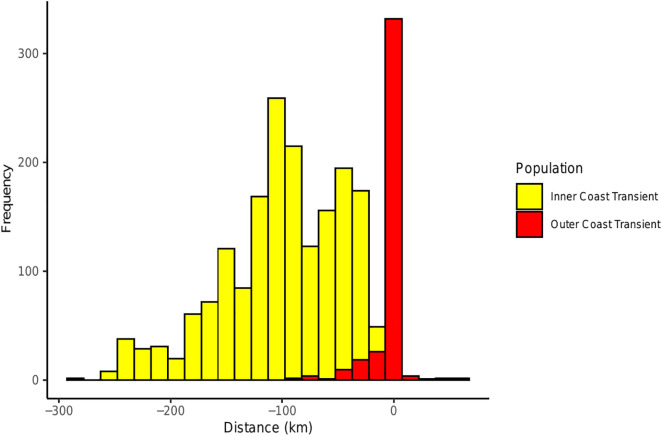
Frequency distributions of encounters with west coast transient killer whales with respect to the distance from the continental shelf break. Negative values indicate distance inshore of the shelf break, and positive values indicate distance offshore of the 200 m isobath where inner coast whales (yellow) and outer coast whales (red) were seen based on 13,152 photo-identifications.

Distance of encounters to the nearest shore differed significantly for the two clusters (Mann-Whitney U test, W = 213, p < 0.05), with inner coast transients occurring an average of 2.4 km from shore (SE = 0.03 km, range 0.001–50 km, n = 11,000 identifications), and outer coast transients occurring 11 km from shore (SE = 0.23 km, range 0.05–121 km, n = 2,152 identifications) (Fig 8). Inner coast transients were also encountered over significantly shallower depths ($\bar{x}$ ± SE = 77.2 m ± 0.68 m, range 1–842 m, n = 11,000 identifications) compared to outer coast transients ($\bar{x}$ ± SE = 347.3 m ± 8 m, range 6–3,572 m, n = 2,152 identifications) (Mann-Whitney U test, W = 196, p < 0.05) (Fig 8). Individuals from the outer coast cluster tended to be closer to the continental shelf break (as delineated by the 200 m isobath) ($\bar{x}$ ± SE = 4.8 km ± 0.25 km, range 1–86.7 km, n = 2,152 identifications) compared to inner coast transients ($\bar{x}$ ± SE = 104.6 km ± 0.52 km, range 1–291.3 km, n = 11,000 identifications) (Mann-Whitney U test, W = 330116, p < 0.05) (Fig 9).

Most encounters with inner coast transients occurred in the protected intracoastal waterways of the Salish Sea where observer effort was highest (Fig 7). This area is frequently traversed by numerous whale watch vessels for much of the year. Similarly, the majority of sightings of outer coast transients occurred off California (particularly Monterey Bay) where the continental shelf is relatively narrow and occurs close to shore, and observer effort is high. However, excluding encounters with both clusters from Monterey Bay and the Salish Sea still showed the same differences in habit use. Both clusters were encountered along the margins of the continental shelf (from southern British Columbia to southern California), with inner coast transients occurring an average of 38.4 km from the shelf break (SE = 0.55 km, range 0–68 km, n = 851 identifications) and outer coast transients occurring an average of just 6.1 km away from the shelf break (SE = 0.55 km, range 0–66 km, n = 432 identifications) (Mann-Whitney U test, W = 339496, p < 0.05).

To determine what drives the association patterns between the two west coast transient killer whale subpopulations, we ran an MRQAP analysis using a symmetric response matrix of associations derived from the killer whale co-occurrence data, and included predictor matrices consisting of pairwise similarities in latitude, longitude, water depth, distance from shore, and continental shelf use. The resulting MRQAP model significantly explained variance in association patterns (adjusted R² = 0.21, F = 23,230, p < 0.001), with the strongest predictors being water depth (β = 0.160, p < 0.001), distance from shore (β = 0.174, p < 0.001), and distance from the continental shelf break (β = 0.068, p < 0.001). In contrast, geographic similarity in latitude and longitude had a small but significant negative effect (β = −0.028, p < 0.001). Overall, the results support the hypothesis that ecological factors such as habitat type and shelf use are more important than geographic proximity in shaping the social associations of the two subpopulations, indicating an outer versus inner coast structuring of relationships rather than a north/south separation of subpopulations.

**Fig 10:**
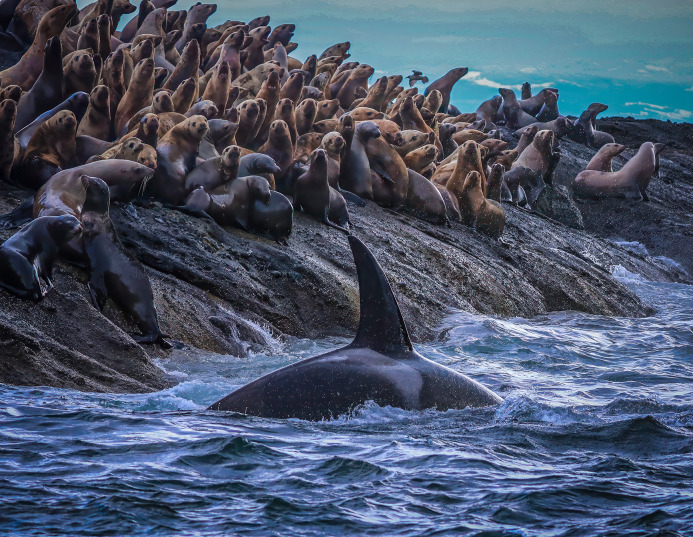
An inner coast transient killer whale patrolling a Steller sea lion haulout off the open coast of Washington. Photograph by Jonathan Scordino, Makah Fisheries Management, MMPA permit 23970.

***Inner coast transient habitat usage***. Movements of inner coast transient killer whales primarily involved combing open waters in straits and bays, and following the contours of coastlines in nearshore waters as they searched seal and sea lion haulouts (Fig 10). The average distance to the nearest shoreline for encounters with inner coast transients was 2.4 km in the Salish Sea (SE = 0.03 km, range = 0.001–15 km, n = 10,117 identifications), 1.2 km off the west coast of Vancouver Island (SE = 0.09 km, range = 0.10–10 km, n = 364 identifications), 3.2 km along the open Pacific coasts of Washington and Oregon (SE = 0.41 km, range = 0.001–50 km, n = 445 identifications), and 5.8 km off the coast of California (SE = 0.72 km, range = 0.8–15 km, n = 63 identifications).

In the protected intracoastal waterways of the Salish Sea, inner coast transients were encountered throughout the Strait of Juan de Fuca, Strait of Georgia, and Puget Sound. Areas frequented by these whales included straits, narrow inlets, open bays, coves, shallow submerged reefs, and rocky shores. Water depths during encounters were highly variable and depended on the location of whales in relation to high relief subsurface bathymetry ($\bar{x}$ ± SE = 81 m ± 0.69 m, range = 1–842 m, n = 10,117 identifications).

Along the exposed open Pacific coast, inner coast transients were observed in similar protected habitats. Off the west coast of Vancouver Island, inner coast whales were encountered in shallow sandy surf zones and inlets that extend far inland ($\bar{x}$ ± SE = 81 m ± 0.69 m, range = 1–842 m, n = 345 identifications). Off the open coast of Washington and Oregon, these whales were observed within meters of sandy surf zones and were frequently documented near open coastal rocky islets in shallow water depths averaging 31.7 m (SE = 3.6 m, range = 1–454 m, n = 445 identifications). In addition, inner coast whales were observed hunting within human-made breakwaters and foraging and capturing harbor seals near rocky intertidal areas, lagoons, and estuaries off the Oregon coast. Similarly, off the coast of California, inner coast whales primarily frequented shallow coastal areas ($\bar{x}$ ± SE = 102 m ± 11.9 m, range = 10–281 m, n = 63 identifications), with encounters occurring close to shore near islets and human-made breakwaters.

**Fig 11:**
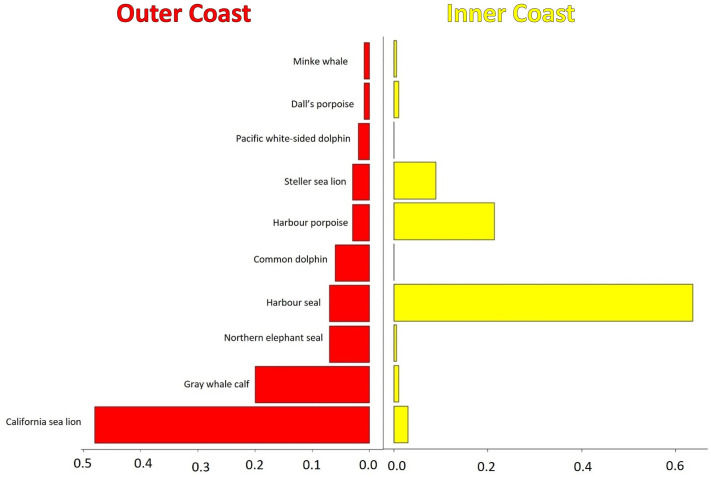
Frequency distribution of recorded predation events by west coast transient killer whales. Data include 201 predation events by inner coast whales (yellow bars), and 98 predation events by outer coast whales (red bars).

Predation events were documented 201 times by inner coast transients at an average of 1.5 km from shore (SE = 0.16 km, range = 0.001–14 km, n = 201 predations) over an average water depth of 70.3 m (SE = 4.8 m, range = 0.51–325 m, n = 201 predations). Approximately three quarters of marine mammal prey consumed were pinnipeds, and one quarter were cetaceans (Fig 11). Harbor seals were the preferred prey, accounting for 64% of total kills. Other pinnipeds consumed included Steller sea lions (9%), California sea lions (*Zalophus californianus*) (3%) and a single northern elephant seal (*Mirounga angustirostris*). Harbor porpoises (*Phocoena phocoena*) were the predominant cetacean prey (21%), with Dall’s porpoises (*Phocoenoides dalli*), minke whales (*Balaenoptera acutorostrata*), and gray whale calves representing <5% of successful kills.

**Fig 12:**
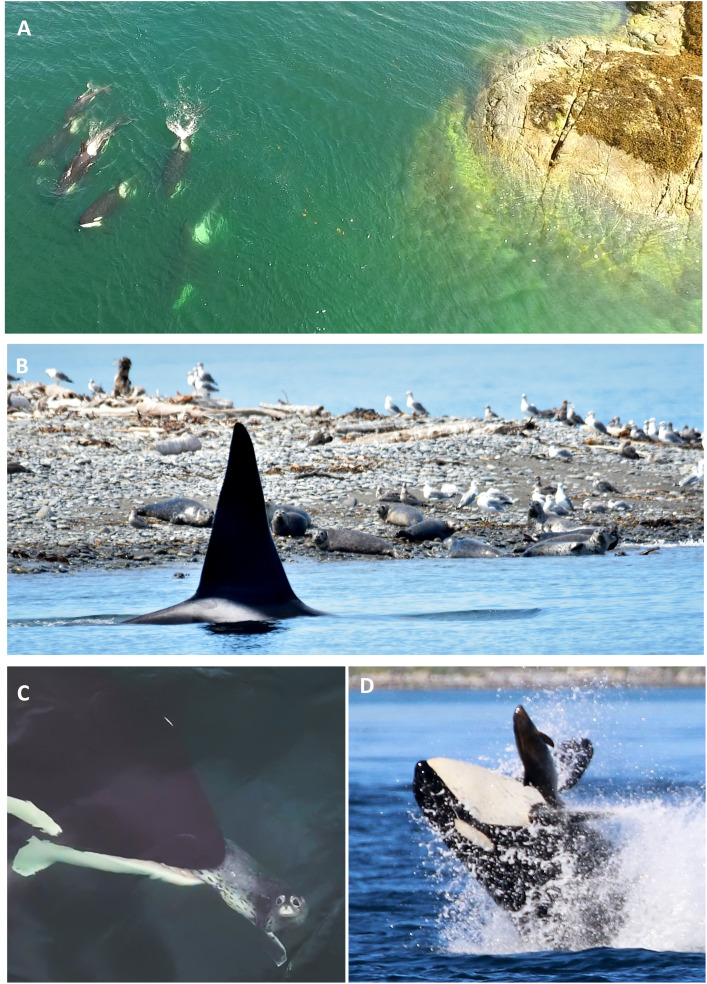
Foraging behavior and predation by inner coast transient killer whales in nearshore waters. **(A)** A group of transients nearshore foraging along an exposed reef in the Salish Sea. **(B)** Adult male transient killer whale T011A patrolling a harbor seal haulout in the Salish Sea. **(C)** A successful capture of a harbor seal pup off the west coast of Vancouver Island. **(D)** Transient killer whale throwing a harbor porpoise into the air during an open water foraging event in the San Juan Islands, Washington State, Photo credits: Josh McInnes **(A)**, Alethea Leddy **(B & D)**, Sydney Dixon **(C)**.

Inner coast transients primarily foraged and captured harbor seals near haulout sites ($\bar{x}$ ± SE = 0.91 km ± 0.08 km, range 0.010–4 km, n = 124 predations). These sites were typically exposed rocky reefs, offshore islets, and rocky or sandy shorelines (Fig 12A). While foraging near a haulout, whales would approach and explore shallow areas and bull kelp beds (*Nereocystis luetkeana*), often surfacing within meters of hauled out seals (Fig 12B). Individual seals were primarily killed beneath the surface or were captured and taken to open water (Fig 12C)—with successful hunts occurring at an average water depth of 60 m (SE = 5.8 m, range = 2.5–325 m, n = 124 predations). The remaining 5 recorded predation events on harbor seals occurred away from haulouts in open water.

In comparison to harbor seals, Steller sea lions were preyed upon much further from shore ($\bar{x}$ ± SE = 2 km ± 0.57 km, range = 0.11–9.2 km, n = 18 predation) and in deeper waters ($\bar{x}$ ± SE = 76 m ± 15.7 m, range = 8.6–243 m, n = 18 predation). Similarly, harbor porpoise predations occurred on average 2.2 km from shore (SE = 0.43 km, range = 0.08–12 km, n = 43 predation) and primarily while whales were foraging in tidal currents or were spread out in open bays, with water depths averaging 90 m (SE = 10.6 m, Range = 2.6–239 m, n = 43 predation) (Fig 12D).

**Fig 13:**
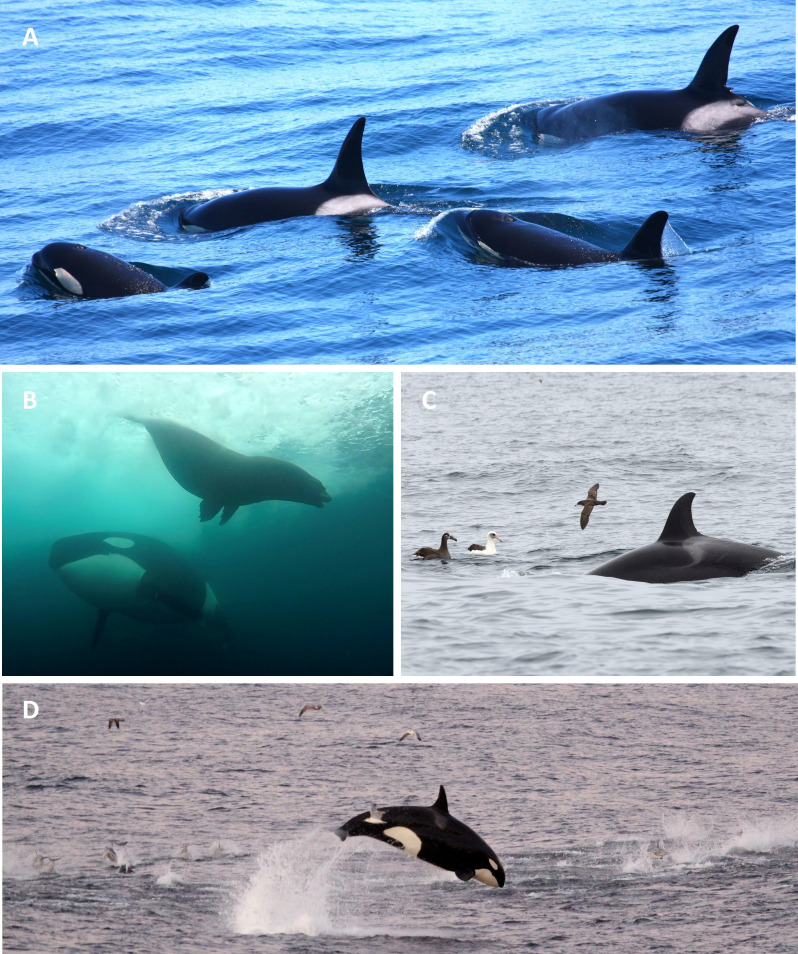
Foraging behavior and predation by outer coast transient killer whales in offshore waters. **(A)** A group of transients following the shelf-break of a submarine canyon in offshore waters of Oregon. **(B)** A female outer coast transient killer whale pursuing an adult male California sea lion in Monterey Bay, California. **(C)** A juvenile outer west coast transient diving to feed on a northern elephant seal carcass, while a black footed albatross (*Phoebastria nigripes*), Laysan albatross (*Phoebastria immutabilis*), and a sooty shearwater (*Ardenna grisea*) wait for scraps. **(D)** An adult female outer coast transient killer whale charging into a school of common dolphins in the open ocean waters off Southern California. Photo credits: NOAA SWFSC **(A, D)**, Stephanie Marcos, Marine Life Studies **(B)**, Amanda Urena, Discovery Whale Watch **(C)**.

***Outer coast transient habitat usage.*** Outer coast transient killer whales were widely distributed in offshore waters, with the majority of encounters occurring along the continental shelf break and near submarine canyons, subsurface seamounts, and offshore islands (Fig 13A). Distance to the continental shelf break was on average 2.4 km off California (SE = 0.51 km, range = 0–53 km, n = 31, shelf width <1–40 km), 22.7 km off the outer coast of Vancouver Island to Oregon (SE = 0.51 km, range = 0–53 km, n = 31, shelf width 15–75 km), and 39.8 km within the intracoastal waterways of the Salish Sea (SE = 2.1 km, range = 3.2–86 km, n = 122, shelf width 3–300 km).

Off California, outer coast transients were photo-identified at 13 submarine canyons, with most resightings occurring at the Monterey, Bodega, Pioneer, Sur, and Lucia canyons (n = 1,739 identifications). Whales were generally observed searching for prey by following the contours of the shelf break and slope, regardless of canyons, in water depths averaging 359.4 m (SE = 7.7 m, range = 19–1637 m, n = 1,729 identifications). Outer coast transients were also documented near the Farallon and Channel Islands, but were primarily observed in open water away from shorelines ($\bar{x}$ ± SE = 19.4 km ± 0.86 km, range = 0.23–50.5 km, n = 250 identifications).

Along the west coast of Vancouver Island, Washington and Oregon, outer coast whales were also observed near the continental shelf break ($\bar{x}$ ± SE = 20 km ± 2.9 km, range = 0–52.8 km, n = 39 identifications) and over deeper waters of the continental slope at an average depth of 822 m (SE = 168.6 m, range = 66–2856 m, n = 39 identifications). Outer coast transients that were previously encountered at a number of submarine canyons off California were also photo-identified at Gray’s, Quinault, Willapa, and Astoria canyons.

In the protected coastal waters of the Salish Sea, encounters with outer coast transients were limited to the Strait of Juan de Fuca. Most encounters occurred in open water and over water depths averaging 102.6 m (SE = 5.21 m, range = 6–187 m, n = 135 identifications) (Fig 7). However, individuals were also observed along the eastern edge of the Juan de Fuca Canyon. These encounters in the Salish Sea were for short periods (<2 days) that ended abruptly when individuals returned to the open ocean.

Predation events by outer coast transients were documented 98 times and occurred an average of 5.2 km from the continental shelf break (SE = 1.4 km, range = 0–97 km, n = 98 predations) in water depths averaging 323.3 m (SE = 34.5 m, range = 7.2–1915 m, n = 98 predations). Approximately two-thirds of marine mammal prey consumed were pinnipeds, and one-third were cetaceans (Fig 11). Pinnipeds consumed were primarily California sea lions (49% of total kills) and northern elephant seals (7%). Gray whale calves were the predominant cetacean prey (20%), but were only observed as prey during spring when whales frequented the Monterey Submarine Canyon System. Other cetaceans consumed included Pacific white-sided (*Lagenorhynchus obliquidens*) and common (*Delphinus delphis*) dolphins (8%).

Predation on California sea lions primarily occurred while whales foraged along the continental shelf break ($\bar{x}$ ± SE = 3.32 km ± 0.80 km, range = 0–28.7 km, n = 48 predations) over water depths averaging 282.4 m (SE = 49.4 m, range = 11.2–1915 m, n = 48 predations) (Fig 13B). In comparison, predation on northern elephant seals (n = 7 predations) occurred over the continental slope ($\bar{x}$ ± SE = 1.63 km ± 0.46 km, range = 0.42–3.5 km) in deeper pelagic waters than used by sea lions ($\bar{x}$ ± SE = 506.7 m ± 152.4 m, range = 115.2–1280 m) (Fig 13C). Both California sea lions and northern elephant seals were attacked in offshore open waters ($\bar{x}$ ± SE = 11.9 km ± 1.6 km, range = 4.26–16.7 km, n = 55 predations). Outer coast transient killer whales were never observed hunting these species near known pinniped haulouts or rookeries (e.g., Farallon Islands, Channel Islands).

Predation on Pacific white-sided and common dolphins (n = 10 predations) were observed close to the continental shelf break ($\bar{x}$ ± SE = 2.81 km ± 0.77 km, range = 0.6–6.2 km) in deep waters ($\bar{x}$ ± SE = 353.5 m ± 91.5 m, range = 45.6–689.1 m). The killer whales primarily searched for these small cetaceans by spreading out over large distances, with members of the group conducting long asynchronous dives along the continental shelf break and slope. Once dolphins were located, the group would initiate a high-speed coordinated pursuit to separate individual dolphins from the larger herd (Fig 13D). Predation events involving common dolphins were only documented off California, while hunts on Pacific white-sided dolphins were spread out in offshore waters throughout each area except the Salish Sea.

A large number of predation events involved gray whale calves as they migrated with their mothers north along the central coast of California during the Spring (n = 20 predations). In California, outer coast transients appeared to use the narrow continental shelf break and slope of submarine canyons (i.e., Monterey, Bodega) to ambush female gray whales and their calves. Distance to the continental shelf break during these hunts averaged 2.12 km (SE = 0.38 km, range = 0.42–5.5 km) and occurred over water depths averaging 425.5 m (SE = 90 m, range = 7.251170 m).

### Association patterns

Out of 2,232 encounters, mixed associations between inner and outer coast transients occurred just 16 times (<1% of all encounters) in areas where the habitat preferences of the two populations overlapped. Of these, 13 different inner coast matrilineal groups, comprising 63 whales (18% of observed population) were photo-identified, while 7 outer coast matrilineal groups, comprising 28 whales and 12 single individual whales were photo-identified (13% of observed population).

The majority of mixed associations occurred in the Strait of Juan de Fuca (n = 13 encounters), and involved brief periods where groups travelled together in open water. Of the 13 encounters, 5 involved observations of different inner coast whales associating with the same outer coast maternal group. The remaining 8 encounters were with different whales from both clusters. However, during these encounters, whales from both clusters primarily associated within their respective clusters, but were within ~200 m of each other. The largest mixed association occurred 15 km off the northern California coast where the continental shelf break was close to shore and the water depth was 205 m. A total of 36 different whales were photo-identified, including 17 inner coast and 19 outer coast transients. Similar to the encounters in the Salish Sea, individuals primarily travelled with members within their respective clusters.

### Geospatial patterns of habitat use

**Fig 14:**
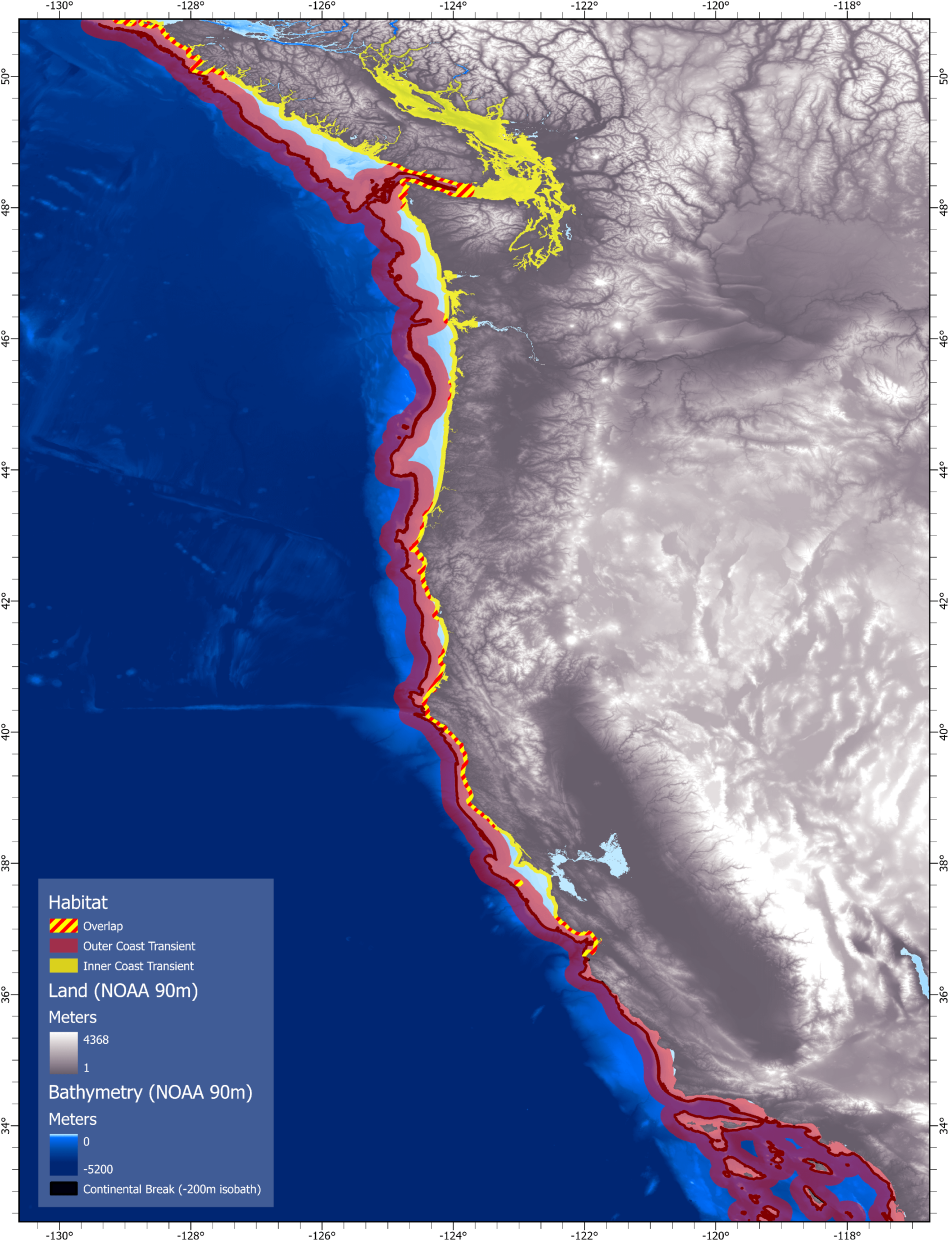
Geospatial map of the relative habitats of inner and outer west coast transient killer whales in the California Current System. This area includes southern British Columbia to southern California. Individuals within the inner coast subpopulation (yellow) were encountered 90% of the time within 5.65 km from shore. Individuals within the outer coast subpopulation (red) were primarily encountered 20 km on either side of the continental shelf-break. Overlap between both subpopulations occur primarily where the continental shelf narrows and approaches close to shore. Relative water depths are shown with shades of blue, with the continental shelf break (200 m) isobath represented by a black line. Base map reprinted from NCEI under CC BY license 4.0, with permission from NOAA, original copyright 2022.

The geospatial analysis of encounters with individual photo-identified WCT killer whales discriminated the habitats of the two clusters (Fig 14). Based on distance from shore, distance from the continental shelf break and water depth data, we mapped the nearest distance to shore as inner coast transient habitat, and mapped distance to the continental shelf break as outer coast transient habitat. Our simplistic habitat model predicts a 90% probability of encountering inner coast transients within 5.6 km of shore, and 90% probability of encountering outer coast transients within 20 km of the shelf break. Overlapping habitat for the two clusters primarily occurs in regions where the continental shelf break approaches the shoreline in the Strait of Juan de Fuca, along the southern Oregon coast, and along the northern and central coast of California (Fig 14).

## Discussion

### Distribution and habitat use

Social network and geospatial analysis distinguished two subpopulations of west coast transient killer whales based on ecological factors (i.e., inner vs. outer coast) rather than latitude (i.e., southern vs. northern territories). The habitats used by the inner and outer coast transient killer whales were best defined by bottom bathymetry and water depth—with overlap most notably occurring in transition zones from shallow to deep waters relative to the distance from the continental shelf break. Observations made during encounters with members of each subpopulation also point to their habitat preferences being closely tied to specific water depths, habitat features, and prey assemblages.

***Inner coast transient killer whales.*** Inner coast transient killer whale habitat use throughout the California Current System (from southern British Columbia, 50° N, to the central coast of California, 36° N) was most consistently associated with proximity to shore, suggesting a potential ecological or behavioral preference for nearshore environments. Our habitat model showed that the core areas used by this subpopulation lie within 5.6 km of the shoreline within intracoastal waterways and along the coastal margins of the continental shelf. The distribution of inner coast transients is primarily continuous, with individual whales being frequently resighted across hundreds of kilometres of coastline. A notable example is the adult female T065B and her maternal group that traveled between the Strait of Juan de Fuca and Monterey Bay, California—a straight-line distance of approximately 1,470 km completed in just 14 days. During this time, they remained within 5 km of shore, hunting harbor seals near coastal rocky islets and near river mouths, as well as preying on two gray whale calves off central Oregon.

The preference for shallow coastal habitats by inner coast transient killer whales aligns with findings from studies across the North Pacific, including Southeast Alaska [7,22], coastal British Columbia [1,2], and the protected waters of southern Vancouver Island and Washington State [12–14,66]. Within the California Current System, members of the inner coast subpopulation were typically observed within 2.4 kilometers from shore in waters averaging 77.2 meters deep. This pattern aligns with observations in British Columbia, where encounters occurred within 2.1 kilometers of shore in waters averaging 98 meters deep [2], and in Southeast Alaska, where transient killer whales generally occurred within 2 kilometers of shore and in varying water depths [7].

The preponderance of inner coast transient encounters in nearshore areas might initially seem to reflect a failure to search for them in offshore regions. However, dedicated surveys covering 49,180 kilometers of offshore areas did not detect inner coast transients beyond 10 kilometers from shore. Opportunistic sightings similarly showed just 3% of the inner coast whale sightings contained in our photoidentification database were more than 10 kilometers from the coast. Thus, inner coast transients appear to have a distinct preference for shallow nearshore habitats over deeper offshore regions.

The distribution and large-scale habitat use patterns of inner coast transient killer whales are likely influenced by the behavior, distribution, and seasonal availability of their preferred prey. Harbor seals were the predominant prey, comprising 64% of observed hunts across the region. This is consistent with previous findings showing harbor seals making up 53% of transient killer whale diets in British Columbia [67], and accounting for 95% of hunts in the protected nearshore waters of the Salish Sea [12]. The diversity of other species consumed by inner coast transients was generally consistent across most of the study areas, except along the Oregon coast, where successful predations on gray whale calves occurred.

Harbor seals are among the most abundant marine mammals along the coast from southern British Columbia to central California. Their populations, however, vary significantly, with densities peaking in northern regions and tapering off further south [17,68]. For instance, the Salish Sea, which hosts an estimated 50,000 harbor seals [68,69], also had the highest diversity of photo-identified inner coast transient killer whales, with a total of 317 individuals recorded. In stark contrast, the west coast of Washington and Oregon supports a significantly smaller harbor seal population, estimated at 11,000–12,000 seals [68,70]. Consequently, our study identified only 110 distinct inner coast killer whales in these areas. This difference in harbor seal abundance along the Pacific coast likely accounts for the lower numbers of photo-identified inner coast transient killer whales observed in the southern regions.

Harbor seals exhibit a unidirectional seasonal cline in the timing of pupping, with weaning and post-weaning occurring later as you go north from California to southern British Columbia [71,72]. These reproductive patterns also likely influence the distribution of inner coast transient killer whales in the California Current by prompting movements to areas with higher seasonal abundances of seal pups. Although a detailed temporal analysis of the relationship between transient killer whale spatial habitat use and prey distribution was beyond the scope of our study, there is suggestive evidence that the seasonal occurrence of inner coast transients is closely linked to the availability of harbor seals [12–14,66,73,74]. For instance, of the 110 photo-identified inner coast transients documented off the Oregon coast, 60 were regularly observed from April through June, coinciding with the pupping season in that region [70,75]. As the pupping season shifted north to the Salish Sea from July through September, 55 of these same whales were consistently resighted in this region. A more detailed analysis of key habitat covariates and seasonal occurrences for inner coast transient killer whales along the Oregon coast would offer further insights into their foraging ecology.

Environmental factors such as the distance to shore, water depth, and proximity to complex bathymetric features—including open straits, intricate inlets, sheltered bays, islets, rocky shorelines, and submerged or exposed reefs—influenced fine scale habitat selection of inner coast transient killer whales. These bathymetric features create habitat in the form of haulout sites for harbor seals and Steller and California sea lions [76,77] and enhance coastal circulation and tidal currents that support harbor porpoise feeding [78]; thus offering productive foraging areas for inner coast transients [7,12,14,77].

Fine-scale habitat differences significantly influence the foraging behaviors of inner coast transient killer whales. In the Salish Sea, these whales are often seen hunting in shallow waters along shorelines as well as over deeper waters in open straits. They frequently followed the contours of shorelines, crossing deeper open water only to navigate to the opposite sides of channels or to patrol pinniped haulout sites. This behavior mirrors what has been well- established for the foraging behavior of inner coast transients in protected nearshore environments, and likely reflects their strategy for hunting in complex, shallow rocky areas where seals and sea lions may seek refuge in nearshore kelp beds or reef crevices [12,14]. In open water, groups of foraging inner coast whales were observed searching for pinnipeds and small cetaceans by dispersing over large distances. Individual whales performed erratic, asynchronous long dives, using tidal currents to ambush prey such as harbor porpoises.

Along the coasts of Washington and Oregon, inner coast transient killer whales were sometimes observed just beyond the surf zones. This area is characterized by an open, high-energy coastline with a relatively uniform continental shelf, offering limited habitat for pinnipeds. Inner coast whales were primarily observed using these open exposed areas to travel between more productive foraging areas. Scattered rocky islets along the open coast serve as refuges for pinnipeds to haul out on, and likely function as important foraging areas for inner coast transient killer whales. However, a large proportion of encounters involved whales frequenting human-made breakwaters, where they hunted harbor seals as far inshore as estuaries and river mouths. These breakwaters also offer sanctuary for pinnipeds, potentially providing foraging habitats similar to the preferred intracoastal waterway systems that inner coast transients rely on in the Salish Sea to ambush and corner prey. Additionally, by foraging along these breakwaters, inner coast whales may avoid the disruptive sounds of the surf zone along the open coast, which could otherwise hinder their ability to passively hear their prey [79].

***Outer coast transient killer whales.*** Outer coast transient killer whales are predominantly found within 20 km of the continental shelf break, frequently near submarine canyons. This subpopulation has a broad distribution with individuals travelling over extensive offshore distances. For example, during a one-month period, the adult female OCT019 and her maternal group were tracked from the Channel Islands, California to the northwest coast of Vancouver Island, BC—covering a straight-line distance of over 2,000 km. They remained within 10 km of the shelf break, hunting northern elephant seals on the continental slope, and preying on a Pacific white-sided dolphin over a Washington submarine canyon.

The habitat preferences of outer coast transient killer whales are not as well-known as those of the inner coast subpopulation due to the challenges of surveying offshore waters. Challenges include unpredictable weather conditions, the remoteness and vastness of oceanic environments, and the high costs associated with operating research vessels in offshore waters. Our results are generally consistent with previous observations from British Columbia, but with some notable differences [2]. In the California Current, encounters with outer coast transients were approximately 11 km from shore and 4.8 km from the continental shelf break, in waters averaging 347.3 meters in depth. Conversely, in British Columbia, they have been typically encountered 8.5 km from shore and 28.8 km from the shelf break, in waters averaging 247.6 meters deep [2]. Both studies indicate a mainly offshore distribution, but these findings indicate that the outer coast whales tend to concentrate in closer proximity to the continental shelf break. The shelf break is generally further from shore in British Columbia (~50 km offshore of Vancouver Island) and narrows to a few kilometers in parts of Oregon and California [37,38,40]. Surveys in British Columbia, have also primarily occurred within 10 km of the coast, with the furthest encounter with transients occurring 30 km offshore [80]; whereas in our study, ship-based surveys covered up to 556 km offshore from southern California to southern British Columbia with the most distant encounter at 121 km offshore [5,50]. Thus, differences in reported distances where outer coast transients have been commonly found likely reflect the extent of offshore coverage, with British Columbia surveys having generally covered areas closer to shore and further from the continental shelf break than surveys in our study within the California Current System.

Occasional sightings of outer coast transient killer whales near coastal regions of British Columbia and Washington State might initially suggest this subpopulation has a broad distribution with no specific preference for offshore waters. However, these few nearshore encounters have occurred where the continental shelf approaches close to shore and near coastal submarine canyons (such as the Juan de Fuca Canyon). Nearshore encounters with outer coast transients have been extensively documented along the California coast, where the continental shelf narrows and intersects nearshore with several large submarine canyon systems [5,15]. Thus, while distance to shore influences certain aspects of outer coast transient habitat, the primary determinant driving their habitat preference appears to be the complex geomorphological and oceanographic features of the continental shelf slope that supports the offshore marine mammal species that these whales specialize on.

The currents and upwelling created by the steep bathymetry of the continental slope and submarine canyons distribute nutrients and enhance productivity that supports marine mammals in open ocean ecosystems [34,43,81–83]. Some of these species include pelagic pinnipeds, oceanic delphinids, and large cetaceans—all of which are targeted by outer coast transient killer whales [15]. Fronts and upwelling events along the continental shelf break entrain zooplanktivorous fish and mesopelagic cephalopods that attract California sea lions and northern elephant seals [84–87]. In some cases, localized productivity can be further enhanced by complex water circulation patterns within submarine canyons that intersect the continental shelf break, where California sea lions and northern elephant seals may preferentially feed [84,86,88,89].

California sea lions made up 49% of the observed marine mammal kills by outer coast transients, underscoring their status as a preferred prey species. This preference is likely due to their large population numbers in the California Current, which averages around 300,000 individuals [17,90]. These pinnipeds breed at rookeries in the Channel Islands from late June to early August. During this period, adult males leave the rookeries in July and migrate northward in the fall and winter, while females remain with their pups but travel to offshore waters to forage [91]. Satellite telemetry has revealed that female sea lions primarily forage along the 500-meter isobath, focusing their activities within 60 kilometers of the rookeries [92]. Satellite telemetry studies of tagged male California sea lions in the Columbia River show movements of individuals throughout continental shelf waters and along the continental slope [93]. In our study, observations of outer coast transients hunting along the 200–500-meter isobath suggest that these whales may utilize this depth zone to intercept California sea lions as they depart from the coast to offshore foraging grounds.

### Separate populations or subpopulations?

In ecological studies, the terms *population* and *subpopulation* are used to describe different aspects of a species’ population and social structure. A *population* refers to a group of individuals of the same species living in a particular area and capable of interbreeding. In contrast, a *subpopulation* is a smaller, distinct group within the larger population, often geographically separated, with unique environmental conditions, or exhibiting distinct behavioral and habitat preferences [94].

Our analysis of association patterns over a 16-year period reveals that WCT killer whales live in a modular, spatially differentiated society consisting of two cohesive subpopulations. These findings support and extend previous conclusions on the spatial segregation of WCT killer whales [2] by incorporating a larger dataset of encounters with the outer coast subpopulation covering a wider geographic range within the California Current System than previously available. Most notably, our analysis shows differential habitat preferences and identifies nearshore areas of spatial overlap along the outer coast where the two subpopulations infrequently interact.

In terms of behavioral and ecological differences, the results from our study show that inner and outer coast transients occupy different habitats across a broad area (from California to southern British Columbia)—and that they have distinct differences in foraging behaviors and dietary specialties. For example, inner coast transients primarily hunt smaller marine mammals such as harbor seals and harbor porpoises, and forage in small groups averaging 5.2 whales [66]. In contrast, outer coast transients form larger groups, averaging 9.2 whales, and target larger prey such as California sea lions, gray whale calves, and Pacific white-sided dolphins [15]. The larger group size of outer coast transients reflects behavioral adaptations for locating and subduing challenging prey in open ocean environments, while inner coast transients may benefit from hunting in smaller groups to optimize energy intake when targeting smaller, easily located prey in protected coastal habitats [13].

Differences in habitat between inner and outer coast transient killer whales—such as proximity to the coast, continental shelf break, and water column depth—require distinct foraging strategies. Inner coast transients appear adapted to shallower, neritic environments, while outer coast transients operate in deeper, pelagic waters [12,15]. These environmental differences likely affect how each group locates, captures, and consumes different prey species, with foraging behaviors being transmitted through association patterns between whales within each subpopulation [73,95–97]. This cultural transmission may ensure effective hunting techniques and the sharing of ecological knowledge within each subpopulation to help calves and juveniles learn to navigate their particular environments effectively [98]. Thus, inner coast transients would become familiar with the location of harbor seal haulout sites and how to safely navigate nearshore channels to access prey without stranding [12,14]. In contrast, outer coast transients probably rely on knowledge of upwelling zones along the continental shelf, which concentrate prey such as California sea lions and northern elephant seals [15,84,86].

The network analysis of individual associations also showed two distinct social networks, with few if any interactions between the inner and outer coast groups (Fig 6). This distinction is further reinforced by previously documented acoustic differences within the WCT population [25,27]. WCT killer whales have four distinct acoustic dialects—A, B, C, and D—each characterized by specific calls. Dialect groups A and B, which share most call types, have been primarily recorded in the coastal waters of British Columbia, Washington State, and Southeast Alaska, while dialects C and D have been predominantly recorded off the coast of California, extending as far north as Haida Gwaii [25,27]. Inner coast transients appear to be more aligned with dialects A and B, whereas outer coast transients are associated with dialects C and D, a pattern supported by preliminary analyses of recordings from photo-identified whales in both groups of killer whales (J. McInnes, unpublished data).

Ultimately, the decision over whether to consider the two groups of WCT killer whales as subpopulations or distinct populations comes down to gene flow and demographic processes where data are currently limited. Although there are no demographic data to assess whether the two groups of killer whales are independent of each other, the limited genetic research conducted along the Pacific coast from Southeast Alaska to southern California has shown that most transients exhibit the WCT mitochondrial DNA haplotype [23,24,99], indicating a shared heritage. This suggests potential interbreeding. Transient populations sampled in California (n = 22) and Southeast Alaska (n = 31) have also been found to share the same haplotype, ENPT1 [100].

Based on the foregoing, the available data support classifying the inner and outer WCT killer whales as separate subpopulations. They exhibit distinct habitat use and spatial segregation with minimal overlap, and have distinct behavioral and ecological differences related to hunting techniques, dietary specialties, social groupings, cultures, and acoustic dialects [25,101,102]. In addition, their east-west distribution rather than a north-south separation means they occur in geographic proximity to each other with some overlap that allows for occasional interactions. Conclusively determining whether they are distinct populations will require additional genetic and life history data to assess their demographic independence and degree of genetic differentiation.

### Ecological divergence of WCT killer whales

The ecological differences noted between the two spatially and socially distinct subpopulations of WCT killer whales may be due to natural environmental changes or more recent phenomenon related to anthropogenic changes in the ecosystem such as the hunting and culling of marine mammal prey. Historically, social groups of transient killer whales may have had a large spatial niche that allowed them to move freely between coastal and offshore habitats in response to natural changes in prey availability, habitat characteristics, or environmental conditions. Alternatively, commercial removals of key pinniped and cetacean prey species may have resulted in transient social groups moving further distances and shifting their habitats either to offshore or inshore waters to find prey.

From 1900 to 1972, extensive culling and harvesting of harbor seals reduced their numbers by over 80% along the Pacific coast, particularly in British Columbia, Washington, and Oregon [103,104]. Similar declines were observed in Steller and California sea lion populations due to control programs and harvesting [17,77,105–107]. Additionally, northern elephant seals were driven nearly to extinction by commercial sealing for their oil rich blubber in the 19th century off California and Mexico, with the population dropping to fewer than 100 individuals [108]. All of these prey depletions likely had a profound impact on the carrying capacity and distribution of WCT killer whales [80]. These changes in prey availability could have caused a spatial regime shift, dietary divergences, and formations of socially and ecologically distinct subpopulations of WCT killer whales [73,95,101,102,109].

Demographically, mark-recapture analyses have estimated that numbers of WCT killer whales in the coastal waters of British Columbia were initially small in the 1970s, and grew rapidly until the mid-1990s at an annual rate of 6% [80]. This rapid population growth is presumed to be due to the recovery of pinnipeds and immigration of new killer whales into the area. Population estimates across four 8-year periods increased from 24 whales in 1974 (95% probability interval = 11–53), to 84 whales in 1982 (range: 52–146), 169 whales in 1990 (range: 120–253), and 210 whales in 1998 (range: 155–289). Most of these whales were likely part of the inner coast subpopulation.

Growth of the inner coast subpopulation decelerated significantly in the 1990s, dropping to approximately 2% per year. This slow, but continued growth was primarily attributed to calf recruitment rather than adult immigration. By 2006, the WCT population had reached 262 whales and was believed to be approaching carrying capacity (95% probability interval = 180–339; [80]) due to stabilization of harbor seal numbers in the coastal waters of British Columbia and Washington State [68,110,111]. However, growth continued as the inner coast population reached at least 304 whales in 2012, 349 whales in 2019 [31], and 372 whales in 2023 (J. McInnes, unpublished data). Reasons for the ongoing growth are unknown, but may be linked to continued growth of the Steller sea lion population and the large numbers of California sea lions occurring south of Washington State [17,66,107].

In contrast to the demographic data available for inner coast WCT killer whales, detailed studies of the outer coast subpopulation are limited. A minimum of 105 transient killer whales were present off the central coast of California in the 1990s [30], many of which were later classified as outer coast transient killer whales [2,5]. A more recent study identified 150 outer coast transients using the canyons and offshore waters of California and Oregon [5]. Combining these numbers with those from Washington, British Columbia and Southeast Alaska suggests a minimum population of 211 outer coast WCT killer whales.

The reduced time spent looking for killer whales in offshore waters, and the extensive periods between sightings of different outer coast transients, make it difficult to compare the population sizes and demographic trends of the inner and outer coast subpopulations. One set of population estimates from photographically recognizable individuals identified 521 WCT killer whales, of which 304 were classified as inner coast and 217 as outer coast transients [2]. These estimates are consistent with our analysis of 556 WCT killer whales a decade later, of which 345 were classified as inner coast and 211 as outer coast transients. Continued research and increased survey efforts is needed to develop a more comprehensive understanding of the demographic dynamics of both subpopulations.

## Conclusion

Our study highlights the presence of two socially, spatially, and ecologically distinct subpopulations of WCT killer whales within the California Current System. The observed spatial segregation between inner and outer coast subpopulations, along with overlapping regions where their habitats converge, suggests a complex dynamic of partial isolation and limited gene flow. These findings underscore the importance of understanding individual variation in habitat use and social structure to inform future monitoring and management efforts for both subpopulations. Our results also reveal the shortcomings of previous research that has been constrained by limited geographic focus and survey coverage, leaving significant gaps in the understanding of population connectivity and gene flow within the WCT population.

Incorporating broader geographic coverage and advanced methodologies, such as satellite telemetry and large-scale genetic studies, is needed to better capture the movements, foraging behaviors, and gene flow patterns of WCT killer whales. Such studies are critical for refining the understanding of population boundaries, especially in offshore areas, and will provide deeper insights into the fine-scale ecology and behavior of inner and outer coast transients. A more comprehensive approach to studying these subpopulations is needed to define groups within species units for conservation and management purposes that reflect the true ecological and genetic diversity of WCT killer whales in the northeastern Pacific. Based on the behavioral and ecological differences between whales within the WCT population, we propose that members of the inner and outer coast transient subpopulations be considered distinct stocks that should be managed separately.

## Supporting information

S1 Table Summary of sighting records, identifications, and associated environmental and effort data used in the behavioral and spatial analyses (XLSX)

S1 Data Supporting information. (XLSX)
